# Transparency and ongoing communication with participants in brain organoid research: Consensus of an interdisciplinary working group

**DOI:** 10.1016/j.stemcr.2025.102546

**Published:** 2025-06-26

**Authors:** Betty Cohn, Megan Doerr, Pamela Feliciano, Stephanie M. Fullerton, Saskia Hendriks, Soren Holm, Insoo Hyun, Karin Jongsma, Karen M. Meagher, M. Elizabeth Ross, Jason L. Stein, Sharon F. Terry, Katherine E. MacDuffie

**Affiliations:** 1 Institute for Public Health Genetics, University of Washington, Seattle, WA, USA; 2 Sage Bionetworks, Seattle, WA, USA; 3 Division of Genetics & Genomics, Department of Pediatrics, Boston Children’s Hospital, Boston, MA, USA; 4 Department of Bioethics & Humanities, University of Washington School of Medicine, Seattle, WA, USA; 5 NIH Clinical Center Department of Bioethics, Bethesda, MD, USA; 6 Centre for Social Ethics and Policy, University of Manchester, Manchester, UK; 7 Center for Medical Ethics, HELSAM, University of Oslo, Oslo, Norway; 8 Museum of Science, Boston, MA, USA; 9 Center for Bioethics, Harvard Medical School, Boston, MA, USA; 10 Bioethics and Health Humanities, University Medical Center, Utrecht University, Utrecht, the Netherlands; 11 Biomedical Ethics Research Program, Mayo Clinic, Rochester, MN, USA; 12 Center for Neurogenetics, Feil Family Brain and Mind Research Institute, Weill Cornell Medicine, New York, NY, USA; 13 Department of Genetics and UNC Neuroscience Center, University of North Carolina at Chapel Hill, Chapel Hill, NC, USA; 14 Genetic Alliance, Damascus, MD, USA; 15 Treuman Katz Center for Pediatric Bioethics and Palliative Care, Seattle Children’s Research Institute, Seattle, WA, USA; 16 Department of Pediatrics, University of Washington School of Medicine, Seattle, WA, USA

## Abstract

Stem cell-based models of the human brain benefit from biospecimens that can be used for a broad range of future research. But current regulations do not address the desire of research participants to remain engaged beyond initial biospecimen donation. We present practicable strategies for engaging participants while preserving scientific potential.

## Introduction

Brain organoids are self-organizing three-dimensional neural tissues derived from pluripotent stem cells and increasingly used in neuroscience research to model aspects of brain structure and function (Kelley and Paşca, 2022). Most commonly, brain organoids are developed from induced pluripotent stem cells (iPSCs) that are reprogrammed from donated biospecimens (e.g., fibroblasts, blood, and urine), which enables the study of how individual variation (i.e., donor genotype) relates to specific neural phenotypes. Individuals, or their proxies, who contribute biospecimens for such work often provide consent to use their cells for unspecified future research (e.g., Department of Health and Human Services [HHS], 2018). Informed consent for the provision of biospecimens occurs at a single time point, yet the iPSC lines have the potential to be used for future research indefinitely, prompting scholars and professional groups such as the International Society for Stem Cell Research (ISSCR) to recommend consent form language that adequately communicates the open-ended nature of iPSC research to potential participants and the likelihood of wide sharing of the resulting iPSC lines across many labs (Lovell-Badge et al., 2021).

In qualitative interviews, participants in brain organoid research have articulated a basic comfort with the notion of one-time consent to cover a wide range of future research (MacDuffie et al., 2023). However, participants also reported a strong desire for ongoing communication with research teams to stay informed about the research developments stemming from their participation. This type of engagement may be particularly important for brain organoid research due to the ever advancing nature of the technology coupled with the ethical and emotional salience of the brain—an organ closely associated with identity and consciousness (Hyun et al., 2020). The historical example of Henrietta Lacks and her family illustrates that some people imbue biospecimens with deep meaning, and long-term research use without consent can have reverberations for personal identity and legacy (Wolinetz and Collins, 2020). Current research guidelines forecast that future developments in brain organoid technology may raise ethical issues that require additional review and oversight (Lovell-Badge et al., 2021). Yet these same guidelines do not include recommendations for communicating ethically relevant information to participants in brain organoid research beyond the initial informed consent process, and there remains no expectation or infrastructure to support ongoing communication between participants and research teams.

In this paper, we describe the deliberations and consensus of an interdisciplinary Working Group that was convened to propose practicable strategies for increased transparency and ongoing communication with participants in brain organoid research.

## Methods

With funding from the National Institutes of Health (NIH; R00MH125328), we convened an interdisciplinary Working Group of 11 scholars representing the disciplines of bioethics, human genetics, laboratory/stem cell research, community engagement, and citizen science. Members were invited based upon their experiences conducting brain organoid research, contributing to stem cell, organoid, or genomic research policy, designing innovative models of research governance or community engagement, and communicating research results to participants.

The group met virtually six times from April to September 2024 and discussed topics related to informed consent, return of results, models of research governance, and infrastructure for ongoing communication. Group members with professional roles that allowed for external compensation received honoraria for their participation.

The Working Group’s aim was to deliberate and reach consensus on a set of strategies to increase transparency during informed consent and augment communication with participants in brain organoid research. We used a combination of structured discussions and an online survey to ensure that all group members’ perspectives were captured and weighed equally. Group consensus was operationalized as >80% agreement (9/11) on survey statements (see Tables S1 and S2). The first and last authors designed the survey and facilitated Working Group discussions. They did not contribute their opinions as part of the consensus survey but were involved in drafting and revising the resulting recommendations.

To determine whether Working Group recommendations aligned with participant preferences, the same online survey was administered to a 7-member participant advisory group, referred to as the Organoid Neuroethics Advisory Panel (ONAP). This 7-member group had been meeting regularly for over 3 years and had previously advised on other aspects of the parent grant (R00MH125328), including creation of an educational video describing brain organoid research, and thus was familiar with the scientific concepts involved. All ONAP members were participants in iPSC/brain organoid research and were either personally affected individuals or the caregiver of a child affected by a neurodegenerative or neurodevelopmental condition. ONAP members were compensated annually via grant funds for their efforts.

In the following paragraph, we describe the major issues discussed by the Working Groups, areas of consensus and dissensus, and note a few areas where the perspectives of the Working Group and ONAP diverged. In crafting recommendations (Table 1), the Working Group attempted to balance competing considerations (e.g., maximizing participants’ engagement/interests, scientific progress, and limited resources) and focused on areas in which we felt that additional standards for brain organoid research are warranted.

**Table 1:** Working Group consensus recommendations

Domain	Recommendations
Transparency in the informed consent process for newly obtained biospecimens	• researchers planning to use iPSC lines to create brain organoids should describe what brain organoids are in the consent form. • researchers should clearly describe plans for sharing data and iPSC lines, articulate a specific timeline for sharing, and clarify the associated implications for consent withdrawal.
Tracking of limitations in secondary uses based on consent	• if specific consent preferences are elicited (e.g., opt out of research with nonhuman animals), there should be standardized metadata approaches for tracking these restrictions that are associated with persistent identifiers when iPSC lines are shared across labs/institutions
Researcher roles and institutional infrastructure for ongoing communication (see also Table 2)	• research teams should consider (1) whether sharing some individual health related results may be appropriate and (2) planning ahead to have resources available for communicating aggregate results to study participants • if researchers plan to return individual or aggregate results, participant preferences for future contact should be elicited during the consent process • research teams and institutions should consider engaging with community advisory boards for input to guide results dissemination and future research directions

### Issues and recommendations

#### Regulatory characteristics and scientific practices that contribute to limited transparency and communication

The Working Group identified four characteristics of current regulatory structures and scientific practice that have contributed to the current status quo of limited transparency with few mechanisms for ongoing communication with contributors of biospecimens for brain organoid research. We describe regulations specific to the United States, which differ in important ways from other jurisdictions (e.g., the European Union) in that almost all biomedical data, including genomic data, can be considered “de-identified” if all direct/indirect identifiers are removed (see Table S3 for specific US regulatory definitions). First, US regulations allow for informed consent for biospecimen donation with few disclosure requirements about the specific research that will be undertaken with de-identified samples and no expectation for ongoing engagement with participants (HHS, 2018). Second, the creation of iPSC lines is expensive and time-consuming, which often leads research groups to outsource steps of the process. Practically, this means that often the researchers consenting and collecting samples are different from those creating the iPSC lines, who might also be different from those differentiating and studying the brain organoids. Third, because biospecimens and iPSC lines are typically de-identified soon after sample collection, most research (after the initial sample collection) with resulting iPSC lines or organoids is not considered “human subjects research” under the Common Rule (HHS, 2018; §46.102). This regulatory distinction means that there is no requirement to consent or otherwise notify the human contributors of the original biospecimens about any aspect of the research including communicating results. An additional implication is that leftover samples from clinical care, if de-identified, can be used for research without obtaining consent (Wolinetz and Collins, 2020). Fourth and finally, the secondary use of de-identified iPSC lines is very common, with sharing of iPSC lines in large-scale repositories increasingly expected by funding organizations (NIH, 2021), and secondary researchers typically have no access to identifiers or contact information from the original participants.

#### Increasing transparency in informed consent for newly obtained biospecimens

The ethical appropriateness of allowing research use of nonidentified biospecimens without consent has been debated for decades. Prior leaders of the NIH called for a revision to this approach, stating “A genuine culture of respect for research participants demands that they be asked to agree to use of their biospecimens, regardless of identifiability” (Wolinetz and Collins, 2020). While some Working Group members thought current research regulations should be overhauled to require consent for use of identifiable or de-identified biospecimens (particularly as technologies like next-generation sequencing and machine learning challenge the feasibility of de-identification), others felt that such a change would hinder research progress to the detriment of public health. Ultimately, the Working Group opted to anchor the recommendations in the current regulations to make them more relevant to researchers in the short and medium term.

Our first two recommendations therefore focus on increasing transparency of the initial consent that is obtained when collecting new biospecimens for research with the intention to create brain organoids. The Working Group and ONAP each reached consensus on these proposed strategies. Importantly, while these strategies address changes to the written consent document, the Working Group strongly endorsed the notion of informed consent as a process, rather than just a form, encouraging research teams to emphasize these consensus themes in informed consent conversations with potential research participants and revisit relevant themes during any subsequent interactions.

Transparency is a core function of informed consent and serves to make individuals aware that they are being asked to participate in a research study and what that study entails (Kraft et al., 2017). It also conveys respect for persons by avoiding deceit and providing the necessary information for potential participants (or their legal representatives) to exercise an autonomous choice about whether to participate. But how much information, or what level of detail, is required in a consent form to achieve transparency? The Common Rule uses the “reasonableness” standard, requiring that prospective participants are provided with “the information that a reasonable person would want to have in order to make an informed decision about whether to participate, and an opportunity to discuss that information” (HHS, 2018; §46.115). The ISSCR (Lovell-Badge et al., 2021) informed consent template for somatic cell donation for iPSC research suggests that researchers disclose the following potential uses of iPSC lines: for gene editing, creation of organoids, transplantation into non-human animals, and (if applicable) planned research involving the creation of gametes and/or embryos. We agree with these recommendations and add that researchers planning to use iPSC lines to create brain organoids specifically should describe brain organoids in the consent form. This recommendation is based upon discussions of the Working Group, input from ONAP, and empirical work with potential and current brain organoid research participants (e.g., MacDuffie et al., 2023) that suggests a proportion of the general public feels that brain organoid research is ethically sensitive and thus warrants specific disclosure. This recommendation applies only to informed consent for newly obtained biospecimens; reconsenting the original participants for all existing iPSC lines currently in use for brain organoid research is not feasible, and requiring such reconsent would stymie important ongoing research—an outcome that both the Working Group and ONAP wished to avoid.

We recommend that research teams planning for specimens to be used to culture brain organoids should specifically describe what brain organoids are (and what they are not—i.e., not full models of the brain) in the consent process. For example, in prior work, a subset of the author team (K.E.M. and J.L.S., with additional colleagues) developed a 5-min animated educational video describing brain organoids for lay audiences, which could be shown to potential participants as part of the informed consent discussion (https://www.seattlechildrens.org/research/centers-programs/bioethics/our-labs/macduffie-lab/projects/#brain). An accessible description of brain organoids could be accompanied by reasons why people might or might not want their samples to be used for brain organoid research (Kraft et al., 2017).

A second recommendation for increased transparency in informed consent is that researchers should clearly describe plans for sharing data and iPSC lines, articulate a specific timeline for sharing, and clarify the associated implications for consent withdrawal. The NIH has published sample language for informed consent for secondary research with data and biospecimens, which includes statements about consent withdrawal such as “Participating in this study means you agree to share your data and biospecimens. You can change your mind later, but researchers might still use your data and biospecimens if they have already been shared” (NIH, 2022). As the NIH document suggests, this language should be enhanced with study-specific examples that specify (1) when data/biospecimens are likely to be shared, (2) who will have access to them (i.e., open- or restricted-access repository), (3) for how long, and (4) how to withdraw consent before samples are shared as well as after (which could halt further distribution, but acknowledging it would be impossible to withdraw samples that have already been shared).

It is possible that such increased transparency could result in some potential participants declining to participate. One media report of this occurring has already been published (Cepelewicz, 2020). However, some individuals declining research participation should not in itself be considered a bad outcome, but rather an indication of a functional consent process. For some participants, providing broad consent for use and sharing of their samples will be concordant with their values. Others will have more conditional preferences, including those that differ for brain vs. other organoid types or related to the use of non-human animals in research.

#### Consent preference collection and tracking

One model that has been proposed for stem cell research is to move from one-time, static consent to “dynamic consent” in which online communication platforms allow participants to adjust their preferences for accessibility of data and samples over time. The Working Group considered such models but felt that the evidence of the benefits of dynamic consent was not yet sufficiently robust to recommend wide-scale adoption. There was concern about the cost of implementation, the disruptive impact of unpredictable changes in consent preferences and sample withdrawal on research progress, and a prediction that researchers would simply elect not to use iPSC lines linked to dynamic consent in their studies to avoid such disruptions.

Within the current one-time consent framework, the Working Group considered the practice of “specific consent” currently employed by some iPSC/organoid labs. Some consent forms contain check boxes for participants to opt in (or out) of specific future uses such as transplantation into non-human animals, next-generation sequencing, or embryo model creation. Importantly, the Working Group did not reach consensus that specific consent should be standard practice, diverging from the opinions of the ONAP group who unanimously felt that it should be. The arguments in favor of specific consent, from both the Working Group and ONAP, centered around the notion that some prospective participants might have objections to specific uses of their cells (e.g., for animal research) and thus being able to opt out of those uses would allow them to still contribute. Arguments against specific consent related to worries that consent restrictions would reduce scientific value of samples, the difficulty of ensuring compliance with consent restrictions, and concern that checking a box is not a robust (nor necessary stable) indication of values and preferences. However, acknowledging that some labs do currently provide opt in/out options for specific future uses, the Working Group strongly endorsed the need to have standardized metadata tracking of such consent restrictions that can travel with iPSC lines once de-identified and shared. Providing specific consent options without a mechanism for tracking those restrictions to ensure compliance by secondary researchers is misleading and undermines participant autonomy. If specific consent is used, tracking of consent restrictions should be part of an iPSC line’s “digital phenotype”—data and metadata files that are linked to an iPSC line’s persistent identifier and include information about the line’s generation (including information about the donor), characterization, and authentication (Wells et al., 2024). Persistent identifiers and metadata tracking are important components of responsible iPSC stewardship and could help overcome the practical hurdles associated with specific consent, allowing more labs to present options related to potentially controversial future research applications if they so choose (Lovell-Badge et al., 2021). Indeed, the persistent identifier (or “name” of the iPSC line) could even be shared with the participant who donated the original sample, enabling the participant to search for and track use of their line in subsequent publications.

The Working Group did not reach consensus on whether informed consent for biospecimen/cell line use for brain organoid research should have an expiration date beyond which “reconsent” would be required for continued use. We discussed this possibility for research conducted with adult biospecimens, which could expire after a set period (e.g., 5 years), as well as research conducted with pediatric biospecimens, which could expire when the child reaches the age of majority (age 18 in most US states and territories). This marked another area of divergence between the consensus opinions of ONAP and the Working Group. ONAP and some Working Group members were in favor of consent expiration, particularly for pediatric participants, due to a desire to respect the emerging autonomy of young adults who might object to future research with organoids derived from their cells. Others in the Working Group were concerned about the required time/cost and logistics of obtaining reconsent, slowed research progress due to attrition of iPSC lines, the complexities of assessing consent capacity for 18 year olds with neurodevelopmental disabilities, and the risk that requiring recontact would mean that samples from participants who are more difficult to reach—e.g., those who frequently change addresses or phone numbers due to financial instability—would be disproportionately excluded resulting in increased inequity for those underrepresented in biomedical research.

#### Researcher roles and institutional infrastructure for ongoing communication

Our final set of recommendations (Table 2) relates to mechanisms for ongoing communication with individual participants and groups that represent donor interests like participant or Community Advisory Boards (CABs; e.g., our ONAP group). CABs are only one example of highly variable public and community engagement practices utilized by biorepositories, and our recommendations related to CABs can be generalized to other community engagement approaches. Given that the Working Group did not reach consensus on the desirability of updating donor preferences over time (i.e., dynamic consent), the intent of these ongoing communication strategies is to encourage research teams to consider (1) communication of research results back to participants and (2) ongoing consultation with CABs for input to guide dissemination and future research directions.

**Table 2:** Consensus recommendations of the role of researchers and institutions in promoting ongoing communication with participants and community engagement

	Researchers			Institutions		
	Physician researchers	Primary researchers	Secondary researchers	Stem Cell Centers/Departments	Biobanks	IRB, ESCRO, and funders
Share individual health-related results with participants	Yes	–	–	–	–	–
Post lay summaries of research results on a public website	Yes	Yes	Yes	Yes	Yes	–
Share interim reports of research progress directly with participants	Yes	Yes	–	–	–	–
Share published results directly with participants	Yes	Yes	–	–	–	–
Consult with an existing community advisory board	Yes	–	–	–	–	–
Host a community advisory board	–	–	–	–	Yes	–
Encourage investigators to share aggregate results	–	–	–	–	–	Yes
Encourage investigators to engage with relevant CAB or similar group	–	–	–	–	–	Yes

‘Yes’ indicates the Working Group reached consensus that this party is likely to have the capability to engage in a given communication/engagement practice.

Researcher definitions: Physician researchers are those who see patients clinically and engage those same patients in their research (collecting biospecimens, etc.). Primary researchers are those working with identifiable, newly collected samples. Secondary researchers are those working with de-identified samples.

ESCRO, Embryonic Stem Cell Research Oversight committees, which exist at some US institutions. While originally convened to monitor research with embryonic stem cells, most ESCROs now have oversight over other types of stem cell research, including organoid research.

The question of whether to share individual, health-relevant results from research is complex, and a full review of the arguments for/against results disclosure is beyond the scope of this paper (see Ravitsky and Wilfond, 2006). Communicating aggregate results (i.e., overall findings) to research participants is less ethically fraught but presents practical challenges as it requires resources that may have been reallocated to other projects by the time results are ready to be disseminated (MacDuffie et al., in press). Building upon decades of evidence that participants want to learn aggregate results, the Working Group recommended that researchers consider (1) whether sharing some individual health-related results may be appropriate, and, separately, (2) planning ahead to have resources available for communicating aggregate results to study participants—or, if study participants cannot be contacted, to the general public. If researchers plan to communicate results directly to participants, the Working Group agreed that participant preferences for future contact with research teams should be elicited during the informed consent process, including their interest in receiving certain individual and/or aggregate results.

The Working Group discussed the role of different researchers and institutions in communicating results back to participants or to the general public (Table 2). Research teams that have a developed relationship with participants (e.g., in longitudinal research) and those with the capability/training to discuss health-related matters (e.g., physician-researchers) are the best positioned to share individual results, if applicable, and aggregate results directly with participants (Ravitsky and Wilfond, 2006). Primary researchers working with identifiable samples have a higher degree of capability for communicating aggregate results directly to participants compared to secondary researchers working with de-identified samples. When communicating aggregate results, the Working Group discussed the relative merits of sharing interim reports of research progress vs. only published results with research participants and reached consensus that both may be shared due to the often slow pace of research progress, educational value of interim reports, and empirical data suggesting that participants want progress updates along the way (MacDuffie et al., 2023).

Effective communication with the public about brain organoid research requires both avoiding overhyped conclusions that create unrealistic expectations for clinical benefits and avoiding exacerbating public concerns about the moral salience of advancing brain organoid capabilities (Hyun et al., 2020). Engaging with CABs and similar groups to understand how members of the public perceive their research may be particularly helpful for brain organoid research teams. However, the Working Group recognized that most individual research groups lack the resources required to host their own CABs. Biobanks were the institutions considered most likely to have sufficient resources to host a CAB, which could then be available for consultation by individual research teams. Consensus was that physician researchers may have a particular obligation for consulting with existing CABs given the nature of their dual relationship (clinician and researcher) with participants and expectation for longitudinal contact beyond the initial biospecimen donation. The Working Group felt that regulatory bodies, like Institutional Review Boards and Embryonic Stem Cell Research Oversight committees, and funders (e.g., the NIH) should encourage investigators to share aggregate results and to consult with CABs or similar bodies.

## Discussion

The Working Group considered whether the aforementioned recommendations should apply only to research with brain organoids or to all research with iPSCs, organoids, or biospecimens and did not reach consensus. Some Working Group members argued that the perception that brain organoid technology could one day create organoids with a rudimentary sentience is sufficient reason for different recommendations (and potentially different regulations) to apply. It was also argued that the specific characteristics and vulnerabilities of participants in brain organoid research, many who are affected by neurodevelopmental or neurodegenerative disorders, make the research worthy of additional regulatory attention. Working group members who disagreed that different recommendations are needed argued that the same near-infinite potential for future research exists for all iPSC lines, that the possibility of brain organoids attaining morally relevant sentience is unrealistic, and that there could be risks to the future of brain organoid research if differential regulation led members of the public or policymakers to misassign morally salient features to organoids. Others also noted that many of the ethical concerns raised by brain organoid research could also arise in other forms of basic and translational neuroscience, such that any ethical carve-outs might be too narrow in scope and therefore application. For these reasons, the Working Group remained unresolved about the appropriate scope of these recommendations and recognizes this will require further deliberation in future international efforts.

The Working Group’s proposed strategies for increasing transparency in brain organoid research have been echoed by similar deliberative efforts conducted internationally. For example, the HYBRIDA Project, funded by the European Commission, produced a number of recommendations related to ethical engagement with individuals who donate biospecimens for brain organoid research. Some of the HYBRIDA recommendations are consistent with those presented here: for example, HYBRIDA similarly recommended that a required set of metadata be associated with organoids (MIAOU; Minimum Information about Organoids and their Use for Researchers) that includes information about the iPSC provenance including restrictions on use based on donor consent (Chneiweiss et al., 2024a). HYBRIDA also recommended that the informed consent process for initial biospecimen donation should describe that participants will be informed about the results of the study and reuse of their biological samples for future research (Chneiweiss et al., 2024b).

Other recommendations by HYBRIDA go further in the direction of supporting participant autonomy than our Working Group’s consensus. For example, HYBRIDA proposed that consent forms for initial biospecimen donation should contain the TRUSTED (Tissue Research Under Secure Transparent Ethical Donation) questionnaire, which captures whether participants authorize use of their cell lines for generating a number of different organoid types (e.g., heart, lung, intestine, brain/nervous system, embryonic models), transfer into laboratory animals, transfer to other institutions, and duration of sample storage (5, 10 years or unlimited; Chneiweiss et al., 2024a). The Working Group considered these types of “specific” consent checklists and time limits on sample use but, unlike HYBRIDA, did not reach consensus to recommend either.

Though the Working Group included members based outside the US, a limitation of our approach was that it focused narrowly on the US regulatory environment, which handles issues such as what can be considered “de-identified” data differently from other countries. Additional limitations relate to the potential bias reflected in our results based upon membership of the Working Group and ONAP. As mentioned, Working Group members were invited based upon their specific interdisciplinary expertise in bioethics, human genetics, laboratory/stem cell research, community engagement, and citizen science, yet they certainly did not represent all potentially relevant perspectives on this topic, and a group with different composition may have reached different conclusions. Similarly, the ONAP group consisted of individuals currently enrolled in brain organoid research projects and thus is not necessarily representative of the broader population of individuals who could be eligible to donate biospecimens.

## Conclusion

Current regulations and scientific practices are not conducive to participants remaining engaged with brain organoid research teams to learn about research results and gain assurance that research being done with their samples aligns with their ethics and values. Our hope is that the aforementioned recommendations for researchers, institutions, and funders encourage consideration of ways to increase transparency and communication with participants and communities engaged in brain organoid research.

## Acknowledgments

We are very grateful to the members of our Organoid Neuroethics Advisory Panel for their invaluable input and feedback on the issues and recommendations discussed here. The educational video linked in the text was created by Booster Shot Media. Funding was provided by 10.13039/100000025NIMH R00MH125328 to K.E.M. The views expressed are the authors’ own and do not represent the NIH, HHS, or US government.

## Author contributions

B.C. and K.E.M. conceived the idea of the study and led the working group meetings. M.D., P.F., S.M.F., S. Hendricks, S. Holm, I.H., K.J., K.M.M., M.E.R., J.L.S., and S.F.T. attended the working group meetings and completed a survey. B.C. and K.E.M. led the writing of the manuscript, and M.D., P.F., S.M.F., S. Hendricks, S. Holm, I.H., K.J., K.M.M., M.E.R., J.L.S., and S.F.T. provided critical feedback to help finalize the manuscript.

## Declaration of interests

The authors declare no competing interests.

## Supplemental information

Document S1. Tables S1–S3

Document S2. Article plus supplemental information
