# Supplementary material for: Transparency and ongoing communication with participants in brain organoid research: Consensus of an interdisciplinary working group
Source: Stem Cell Reports. 2025 Jun 26;20(9):102546. doi: 10.1016/j.stemcr.2025.102546 (PMC12447313; doi:10.1016/j.stemcr.2025.102546)
Supplement: Document S1. Tables S1–S3 [file mmc1.pdf]

Stem Cell Reports, Volume *20*

Supplemental Information

Transparency and ongoing communication with participants in brain organoid research: Consensus of an interdisciplinary working group

Betty Cohn, Megan Doerr, Pamela Feliciano, Stephanie M. Fullerton, Saskia Hendriks, Soren Holm, Insoo Hyun, Karin Jongsma, Karen M. Meagher, M. Elizabeth Ross, Jason L. Stein, Sharon F. Terry, and Katherine E. MacDuffie

Supplementary Table 1: Delphi Survey Results from the Working Group and ONAP

Question	Working Group	ONAP
Researchers planning to use cell lines to create brain organoids should describe this intention in the consent form.	Agree 11/11	Agree 6/6
Researchers planning to transplant human neural cells into non-human animals should describe this in the consent form.	Agree 9/11	Agree 6/6
A clear timeline for data sharing and implications for sample withdrawal should be communicated (e.g., "after 6 months your cells will be deposited in X repository, after which point you will be unable to withdraw them").	Agree 11/11	Agree 6/6
A donor's preference for future contact with research teams should be elicited, including interest in receiving individual and/or aggregate results.	Agree 11/11	Agree 6/6
Methods should be standardized for tracking consent preferences associated with cell lines when shared across labs/institutions (e.g., whether the original consent allowed for creation of brain organoids, transplant into animals, expiration date, etc.).	**Agree 11/11**	Agree 4/6
Regulations allowing broad consent for biospecimen donation should be modified for BO research.	Yes 3/11	Yes 4/6
Consent for use of biospecimens/cell lines should have an expiration date (e.g., 5 years, or when a child turns 18), beyond which further use of the cell lines is not permitted without explicit reconsent from donor.	Agree 6/11	Agree 5/6
Donors should be able to opt out of consenting to specific future uses of their cells for BO research (e.g., transplantation into animals, other humans, etc.)	Agree 8/11	Agree 6/6
A "dynamic consent" process should be employed that allows donors to track (and potentially change) their consent preferences over time.	Agree 5/11	Agree 3/6
Do you think these recommendations should be different for brain organoid compared to other types of organoid research?	Yes 5/11	Yes 2/6
Do you think these recommendations should be different for organoid research compared to other research using induced pluripotent stem cells (iPSCs)?	Yes 4/11	Yes 2/6
Do you think these recommendations should be different for organoid/iPSC research compared to other research using donated human biospecimens?	Yes 6/11	Yes 2/6

Questions included the following response options: Strongly agree, agree, neutral, disagree and strongly disagree. We grouped strongly agree, agree and neutral into agree when calculating results.
Grey shaded cells indicate where consensus was reached.

Supplementary Table 2: Delphi Survey Results from Working Group

	RESESEARCHERS			INSTITUTIONS				
	Physician researchers	Primary researchers	Secondary researchers	Stem Cell Centers/Depts	Biobanks	IRB	ESCRO	Funders
Share individual health-related results with participants	Agree 91%	Agree 64%	Agree 27%					
Post lay summaries of research results on a public website	Agree 91%	Agree 82%	**Agree 82%**	**Agree 91%**	**Agree 82%**			
Share interim reports of research progress directly with participants	Agree 82%	Agree 82%	Agree 36%	Agree 70%	Agree 55%			
Share published results directly with participants	Agree 82%	Agree 82%	Agree 18%	Agree 60%	Agree 60%			
Consult with an existing Community Advisory Board	Agree 82%	Agree 73%	Agree 64%					
Host a Community Advisory Board	Agree 73%	Agree 73%	Agree 45%	Agree 80%	Agree 82%			
Encourage investigators to share aggregate results						**Agree 82%**	Agree 56%	Agree 100%
Encourage investigators to engage with relevant CAB or similar group						**Agree 100%**	Agree 100%	Agree 100%
Requiring investigators to engage with a CAB						**Agree 82%**	Agree 70%	Agree 80%
Reviewing and approving materials for communicating aggregate results						**Agree 82%**	Agree 67%	
Requiring investigators to share aggregate results						Agree 55%	Agree 56%	Agree 80%
Encouraging investigators to share aggregate results						Agree 82%	Agree 67%	Agree 100%

We present results as percentages for ease of comparison across cells as not all working group members responded to all items.
Grey shaded cell indicate where consensus was reached.
Responses from ONAP members are not displayed as ONAP members were not asked this set of questions.

Supplementary Table 3: Regulatory definitions from the United States Common Rule

Term	Definition	Law
De-identified samples	"Information, which may include information about biospecimens, is recorded by the investigator in such a manner that the identity of the human subjects cannot readily be ascertained directly or through identifiers linked to the subjects, the investigator does not contact the subjects, and the investigator will not re-identify subjects"	Common Rule (45 CFR § 46.104)
Identifiable biospecimen	An identifiable biospecimen is a biospecimen for which the identity of the subject is or may readily be ascertained by the investigator or associated with the biospecimen	Common Rule (45 CFR § 46.102)
Human subjects	Human subject means a living individual about whom an investigator (whether professional or student) conducting research:<br>i) Obtains information or biospecimens through intervention or interaction with the individual, and uses, studies, or analyzes the information or biospecimens; or<br>ii) Obtains, uses, studies, analyzes or generates identifiable private information or identifiable biospecimens.	Common Rule (45 CFR § 46.102)
Research	Research means a systematic investigation, including research development, testing, and evaluation, designed to develop or contribute to generalizable knowledge.	Common Rule (45 CFR § 46.102)
